# Detection of Human CD38 Using Variable Lymphocyte Receptor (VLR) Tetramers

**DOI:** 10.3390/cells9040950

**Published:** 2020-04-12

**Authors:** Srijit Khan, Yanling Liu, Laura M. Ernst, Leslie Y. T. Leung, Patrick Budylowski, Shilan Dong, Paolo Campisi, Evan J. Propst, Nikolaus E. Wolter, Eyal Grunebaum, Mario Ostrowski, Götz R. A. Ehrhardt

**Affiliations:** 1 Department of Immunology, University of Toronto, Toronto, ON M5S 1A1, Canada; srijit.khan@utoronto.ca (S.K.); yanling.liu@utoronto.ca (Y.L.); laura.ernst@mail.utoronto.ca (L.M.E.); leslieyt.leung@mail.utoronto.ca (L.Y.T.L.); shilan.dong@mail.utoronto.ca (S.D.); mario.ostrowski@gmail.com (M.O.); 2 Department of Otolaryngology—Head and Neck Surgery, Hospital for Sick Children and University of Toronto, Toronto, ON M5G 1X8, Canada; paolo.campisi@sickkids.ca (P.C.); evan.propst@sickkids.ca (E.J.P.); nikolaus.wolter@sickkids.ca (N.E.W.); 3 Division of Immunology and Allergy, Hospital for Sick Children and University of Toronto, Toronto, ON M5G 1X8, Canada; eyal.grunebaum@sickkids.ca

**Keywords:** variable lymphocyte receptor (VLR), evolution, tetramer, flow cytometry

## Abstract

CD38 is a multifunctional cell surface receptor expressed on multiple cell lineages of hematopoietic origin with high levels of expression on human plasma cells. Previously, we isolated the monoclonal variable lymphocyte receptor B (VLRB) MM3 antibody from the evolutionarily distant sea lamprey, which recognized the CD38 ectoenzyme exclusively on human plasma cells in a manner that correlated with CD38 enzymatic activity. The plasma cell-specific binding of VLRB MM3 contrasts with the broad pattern of expression of CD38-determined conventional antibodies specific for this antigen. In an effort to facilitate the application of this unique reagent in combination with conventional antibody panels, we explored a strategy to generate VLRB MM3 tetramers. The resulting reagent maintained the threshold-based recognition of CD38. Increased sensitivity achieved with VLRB MM3 tetramers also showed preferential recognition of germinal center centroblasts over centrocytes. VLRB MM3 tetramers thus provided a unique and versatile single-step staining reagent for the detection of human CD38 that is readily incorporated into multi-color flow cytometry panels.

## 1. Introduction

CD38 is an ectoenzyme with nicotinamide adenine dinucleotide (NAD) hydrolase and NAD cyclase activity and is expressed on a variety of cells, including epithelial cells, skeletal and cardiac muscle fibers, and numerous hemopoietic cell lineages [1,2]. Engagement of CD38 on B and T lineage cells influences various cellular responses, ranging from regulation of antigen receptor signaling to the modulation of apoptosis [3,4,5,6]. Expression levels of CD38 on human B cells vary during B cell development and differentiation. It is expressed on B lineage precursors in the bone marrow and following B cell activation on germinal center B cells, but it is expressed only at very low levels on naïve and memory B cells [7,8]. In contrast, it is expressed at high levels on antibody-secreting plasma cells and plasmablasts, making it an immunotherapeutic target of monoclonal antibodies in multiple myeloma [9].

Conventional immunoglobulin-based antibodies derived from jawed vertebrates are invaluable biomedical research reagents due to the specificity with which they recognize selective antigens. This specific antigen recognition is the basis for their increasing importance in clinical diagnostic, prognostic, and therapeutic applications. However, structural and tolerogenic constraints are limiting factors on the antibody repertoire. The evolutionarily distant jawless vertebrates, on the other hand, have an adaptive immune system that utilizes variable lymphocyte receptor (VLR) antibodies with leucine-rich repeat (LRR) as the basic structural unit [10,11]. Three immature VLR genes—VLRA, VLRB, and VLRC—undergo a gene conversion-like maturation process and are expressed in a mutually exclusive manner on cells with similarities to αβ T cells [12], B cells [13], and γδ T cells [14], respectively. Among the mature VLR proteins, only VLRB molecules are found as secreted multimeric complexes [15]. Structural analyses of recombinant VLRB proteins in complex with their respective antigens have shown that VLR proteins assume a solenoid shape, and antibody/antigen interactions occur with residues lining the concave surface in addition to residues forming a flexible loop protruding from the capping C-terminal LRR unit [16,17,18]. In earlier studies, we isolated a monoclonal VLRB antibody from lamprey larvae immunized with multiple myeloma bone marrow aspirate—VLRB MM3—specifically recognizing the CD38 antigen on human plasma cells and plasmablasts [19]. Similar to observations with a CD5-specific monoclonal VLRB antibody [20], recognition of CD38 was observed with decameric VLRB MM3 but not with individual monomeric antigen-binding units [19]. Antigen recognition correlated with CD38 enzymatic activity, and competition experiments with non-hydrolyzable NAD analogs indicated that the epitope recognized by VLRB MM3 was located within the catalytic site of the ectoenzyme [19]. These antigen recognition characteristics make VLRB MM3 a unique reagent in the analysis of human CD38. Using this antibody in applications, such as flow cytometry, ideally requires direct fluorochrome labeling of the protein. However, monoclonal VLR antibodies are not suitable for direct labeling as the commonly used amine- or thiol-reactive labeling chemistry can impede antigen recognition. To address this challenge, we explored the generation of VLR-tetramers assembled from monomeric biotinylated VLRB MM3 units. VLRB MM3 tetramers maintained the unique antigen recognition characteristics of conventional recombinant VLRB MM3 molecules with enhanced sensitivity and were readily applicable for single-step multi-color flow cytometry experimentation.

## 2. Materials and Methods

### 2.1. Cells and Reagents

HEK293T cells were grown in DMEM supplemented with 5% fetal bovine serum, 2 mM l-glutamine, and 100 U/mL penicillin-streptomycin. Human B cell lines Daudi, BJAB, and KMS-11 were grown in RPMI1640 supplemented with 10% fetal bovine serum, 50 μM β-mercaptoethanol, 2 mM l-glutamine, and 100 U/mL penicillin-streptomycin. Cells were grown at 37 °C at 5% CO_2_ in a humidified atmosphere. Antibodies to the CD3 (clone HIT-3a, PerCPCy5), CD19 (clone HIB-19, APC-Cy7), CD38 (clone HIT-2, FITC), IgD (clone IA6-2, BV395), and CXCR4 (clone 12G5, Pe-Cy7) antigens were obtained from BD Biosciences (San Jose, CA, USA). Streptavidin, coupled to phycoerythrin, was obtained from Southern Biotech (Birmingham, AL, USA). D-Biotin was purchased from BioShop Canada (Burlington, ON, Canada), and ATP was obtained from Promega (Madison, WI, USA). Coumermycin and NAD were obtained from Sigma-Aldrich (St. Louis, MO, USA), and β-ara-2′-deoxy-2′-fluoro NAD (araF) from BioLog (Bremen, Germany). The bacterial expression plasmid encoding BirA was a generous gift from Dr. Jean-Philippe Julien (Hospital for Sick Children, Toronto, ON, Canada). Tonsillar tissue from pediatric patients undergoing tonsillectomy was obtained from the Hospital for Sick Children (Toronto, ON, Canada), with informed consent according to the Declaration of Helsinki and the institute’s Research Ethics Board.

### 2.2. Generation of Monomeric VLRB MM3 for Site-Specific Biotinylation

Custom synthesized DNA sequences encoding the HA-epitope tag, 6xHis-epitope tag, and BirA recognition sequence (YPYDVPDYASHHHHHHGLNDIFEAQKIEWHE) were cloned into the pIRESpuro2 eukaryotic expression vector. Monomeric VLRB MM3 antigen-binding units lacking the 56 C-terminal amino acids were generated by a polymerase chain reaction and cloned upstream of the HA-epitope tag. Cloned sequences were validated by DNA sequencing and transfected into HEK293T cells. Monomeric protein expression and secretion into the culture supernatant of transfected cells were validated by western blotting using anti-HA and anti-His antibodies. Transfected HEK293T cells were drug selected using puromycin at 0.5 μg/mL, and secreted recombinant VLR molecules were purified from culture supernatants by nickel affinity chromatography, as described previously [19,21].

### 2.3. Biotinylation of Monomeric Recombinant VLRB MM3

In vitro biotinylation of monomeric VLRB MM3 units was performed using the protocol described in [22]. Briefly, 100 μM of monomeric VLRB MM3 units in phosphate-buffered saline were combined with 50 μM of BirA enzyme in the presence of 5 mM magnesium chloride, 2 mM ribo-ATP, and 0.15 mM D-Biotin. The mixture was incubated at 30 °C for 1 h, followed by the addition of an equal amount of BirA enzyme and ribo-ATP and an additional hour of incubation at 30 °C. Unincorporated biotin was removed by dialysis; the biotinylated protein was then concentrated using SPIN-X UF6 spin columns (Corning Life Sciences, Tewksbury, MA, USA) and frozen in aliquots at −80 °C.

### 2.4. Use of VLRB MM3 Tetramers in Flow Cytometry Panels

Biotinylated monomeric VLRB MM3 units were incubated for 30 min with streptavidin-PE in PBS/0.5% bovine serum albumin (BSA) for assembly into of ready-to-use VLRB-tetramers (tetVLRB MM3) that could be used as a single step staining reagent. Dilutions of the resulting tetVLRB MM3 tetramers were incubated with Daudi or KMS-11 cells for 15 min on ice, followed by two washes with PBS/0.5% BSA. Dead cells were excluded using propidium iodide staining, and the tetVLRB MM3 tetramer binding was assessed using a Guava Easycyte flow cytometer (MilliporeSigma, Burlington, MA, USA). Control experiments using conventional decameric VLRB MM3 were performed as described earlier [23]. For tetVLRB MM3 tetramer staining of primary cells, single-cell suspensions of tonsillar tissue were obtained by tissue mincing using a 70 μm steel mesh, followed by density gradient centrifugation using lymphocyte separation medium (ThermoFisher Scientific, Waltham, MA, USA). The resulting tonsillar mononuclear cell suspension was blocked in 5% normal mouse serum, followed by incubation with an antibody cocktail consisting of tetVLRB MM3 tetramers and anti-CD3, anti-CD19, anti-CD38, and anti-IgD antibodies for 20 min on ice. Cells were washed twice with PBS/0.5% BSA. Dead cells were excluded using the fixable Aqua Live/Dead reagent (ThermoFisher Scientific, Waltham, MA, USA), and data were acquired using a BD LSR-II instrument (San Jose, CA, USA). Flow cytometry data were analyzed using the FlowJo software package (Ashland, OR, USA).

### 2.5. CD38 Dimerization Assays

CD38 dimerization assays were performed, as described previously [19]. Briefly, human BJAB Burkitt’s lymphoma cells were transiently transfected with CD38-GFP-GyrB expression constructs by electroporation (300 V/975 μF). Following electroporation, the cells were incubated for 24 h before the addition of coumermycin (Cm) for 30 min. For blocking experiments involving NAD or β-ara-2′-deoxy-2′-fluoro NAD, various concentrations of the NAD analog or NAD were added in addition to 2 μM coumermycin to induce dimerization of CD38-GFP-GyrB. Subsequently, cells were washed and incubated with tVLRB MM3 tetramers for 20 min on ice. Dead cell exclusion was performed by the addition of propidium iodide (1 μg/mL), and the tetVLRB MM3 binding was assessed using a Guava EasyCyte flow cytometer (MilliporeSigma, Burlington, MA, USA). The tetVLRB MM3 binding data were analyzed using the FlowJo software package (Ashland, OR, USA).

### 2.6. Statistical Analysis

Statistical significance was determined using Kruskal–Wallis, Mann–Whitney U tests, and Friedman test with Dunn’s post hoc analysis as detailed in the figure legends using the GraphPad Prism 6.0 software package (San Diego, CA, USA).

## 3. Results

### 3.1. VLRB MM3 Tetramers Maintain Cell Line Recognition Patterns with Enhanced Sensitivity

**Figure 1:**
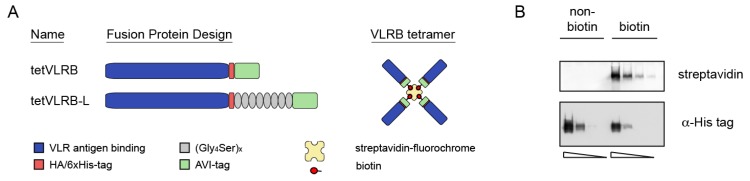
Generation of monomeric variable lymphocyte receptor B (VLRB) for sequence-specific biotinylation. (**A**, left panel) Design of monomeric VLRB MM3 antigen-binding units lacking the C-terminal 56 aa. These residues were replaced with the HA- and 6xHis epitope tags and the BirA recognition sequence (AVI-tag) for enzyme-mediated biotin transfer. (Right panel) Assembled VLRB MM3 tetramer. (**B**) Serial 1:5 dilutions of biotinylated and non-biotinylated tetVLRB MM3 were separated by SDS-PAGE and analyzed by western blotting for biotin incorporation (top membrane) and protein content (bottom panel). Shown is a representative of four independent experiments.

The introduction of major histocompatibility complex (MHC) I tetramers was a milestone that allowed the detailed analyses of antigen-specific T cells during the course of immune responses [24,25]. In order to investigate whether a similar approach was possible for the unique CD38 binding characteristics of VLRB MM3, we designed monomeric VLRB MM3 molecules of which the C-terminal 56 amino acids containing eight cysteine residues involved in disulfide bond formation for VLRB multimerization were replaced by sequences encoding the HA- and 6xHis epitope tags and the recognition sequence for the BirA biotin ligase (tetVLRB MM3, Figure 1A, top). VLRB molecules contain an extended flexible stalk region between the antigen-binding domain and the cysteine-rich region involved in disulfide bond formation. To add potentially needed structural flexibility to VLRB tetramers, we designed a second monomeric VLRB MM3 in which the BirA biotin ligase recognition sequence and the HA- and 6xHis epitope tags were separated by an 8-times repeat of the Gly_4_Ser linker (tetVLRB-L MM3 Figure 1A, bottom). Following biotinylation and incubation with fluorochrome-labeled streptavidin, these individual antigen-binding units assembled into tetrameric complexes (Figure 1A, right panel). As anticipated, monomeric VLRB MM3 protein purified by nickel affinity chromatography could be efficiently biotinylated in vitro (Figure 1B).

**Figure 2:**
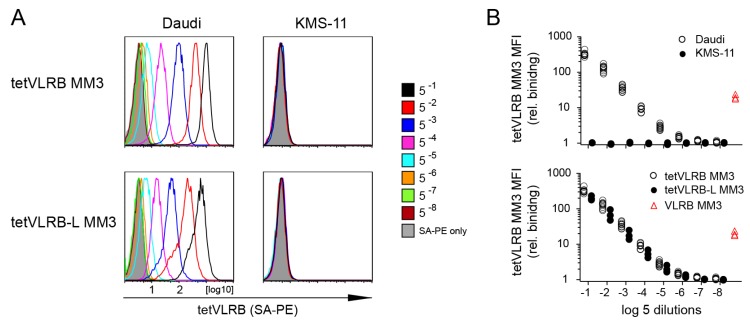
Cell line recognition of tetVLRB MM3 and tetVRB-L MM3 tetramers. (**A**) VLRB MM3-reactive Daudi and VLRB MM3 non-reactive KMS-11 cell lines were incubated with the indicated dilutions of tetVLRB MM3 and tetVLRB-L MM3 tetramers. Negative control experiments (phycoerythrin-labeled streptavidin (SA-PE) only) are indicated by shaded grey histograms. Shown are representative experiments. (**B**) Combined analyses of tetVLRB MM3 tetramer recognition of Daudi (open circles, *n* = 10) and KMS-11 (closed circles, *n* = 3) cell lines (top panel). Combined analyses of tetVLRB MM3 (open circles, *n* = 10) and tetVLRB-L MM3 (closed circles, *n* = 3) recognition of Daudi cells (bottom panel). Red triangles (*n* = 5) indicate optimal staining of Daudi cells using conventional decameric VLRB MM3. Shown are median fluorescent intensity (MFI) values normalized to MFI values obtained in negative control experiments (SA-PE only).

Subsequently, we coupled in vitro biotinylated VLRB MM3 (tetVLRB MM3) and its longer form containing eight repeats of a (Gly_4_Ser) linker (tetVLRB-L MM3) to phycoerythrin-labeled streptavidin (SA-PE), followed by flow cytometric assessment of cell line recognition. Previously, we demonstrated that recombinant decameric VLRB MM3 was reactive with the Daudi Burkitt’s lymphoma but not the KMS-11 plasmacytoma cell lines [19]. These experiments showed that both tetVLRB MM3 and tetVLRB-L MM3 maintained the recognition of Daudi cells but did not react with KMS-11 cells at any of the concentrations of a broad range of dilutions of the reagents tested (Figure 2A,B). Moreover, both tetVLRB MM3 preparations showed great enhancement of recognition sensitivity compared to conventional VLRB MM3 used at optimum conditions to achieve maximum staining intensity (Figure 2A,B). Since tetVLRB MM3 and tetVLRB-L MM3 showed comparable sensitivity, we performed subsequent experiments with the tVLRB MM3 reagent lacking the 8x(Gly_4_Ser) linker sequences.

### 3.2. tetVLRB MM3 Tetramers Recognition of CD38 Is Enhanced Following CD38 Dimerization and Blocked by Non-Hydrolyzable NAD Analogs

**Figure 3:**
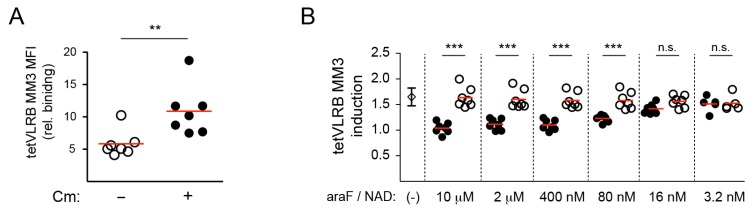
Increased recognition of CD38 by tetVLRB MM3 tetramers following CD38 dimerization. (**A**) BJAB cells transiently transfected with CD38-GFP-GyrB were treated with coumermycin (2 μM) and assessed for tetVLRB MM3 binding. tetVLRB MM3 signals of GFP-positive cells were normalized to MFI values obtained in negative control experiments (SA-PE only). Statistical significance was determined using Mann–Whitney U tests and is indicated as ** *p* < 0.01 (*n* = 6). Red bars indicate mean values. (**B**) BJAB cells transiently transfected with CD38-GFP-GyrB were treated with araF (closed circles) or NAD (open circles) prior to addition of coumermycin (2 μM). Experiments without araF or NAD treatment are indicated by an open diamond symbol (mean ± SD). tetVLRB MM3 binding to transfected GFP-positive cells was normalized to values of cells without coumermycin treatment. Statistical significance for each concentration was determined using Mann–Whitney U tests and is indicated as *** *p* < 0.001, n.s. *p* > 0.05 (*n* = 7, except for lowest concentration *n* = 5).

CD38 exists in three conformations: monomeric, dimeric, and tetrameric, and aggregation of CD38 correlates with its NAD hydrolase/cyclase enzymatic activity [26,27]. Earlier, we demonstrated that conventional decameric VLRB MM3 recognition of CD38 could be enhanced in cells expressing CD38-GFP-gyraseB fusion proteins in which the addition of the coumermycin antibiotic and binding to gyraseB led to dimerization of the fusion protein [19]. Importantly, coumermycin treatment of the cells did not change CD38 expression levels [19]. Similar to observations with conventional decameric VLRB MM3, tetVLRB MM3 binding to CD38-GFP-gyraseB transfected but not untransfected cells could be enhanced following induction of dimer formation of the fusion protein (Figure 3A). Studies using conventional decameric VLRB MM3 showed that binding of decameric VLRB MM3 to CD38 could be inhibited by pre-incubation of the target cells with a non-hydrolyzable analog of NAD [19]. Using the same experimental approach, we observed that the increased recognition of CD38 by tetVLRB MM3 tetramers following the addition of coumermycin was inhibited by pre-incubation of cells with the non-hydrolyzable β-ara-2′-deoxy-2′-fluoro NAD (araF) inhibitor of CD38, but not by pre-incubation with NAD (Figure 3B). These experiments indicated that decameric VLRB MM3 and tetVLRB MM3 tetramers maintained the same CD38 antigen recognition characteristics.

### 3.3. tetVLRB MM3 Tetramers Preferentially Recognize Human Plasma Cells and Distinguish Germinal Center Centrocytes From Centroblasts

**Figure 4:**
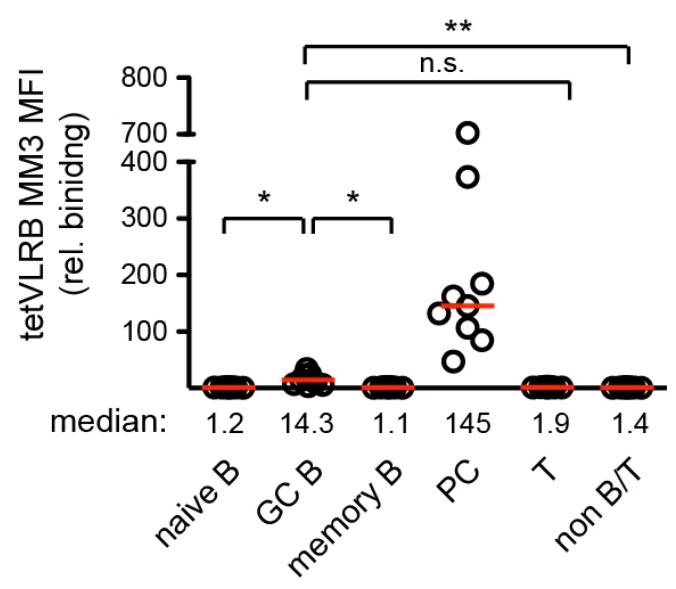
Plasma cell recognition of tetVLRB MM3 tetramers. Tonsillar monocuclear cells were incubated with tetVLRB MM3 tetramers in combination with antibodies recognizing CD3, CD19, CD38, and IgD. Cells were separated into CD19^+^/CD3^−^/CD38^−^/IgD^+^ naive B cells, CD19^+^/CD3^−^/CD38^+^/IgD^−^ germinal center (GC) B cells, CD19^+^/CD3^−^/CD38^−^/IgD^−^ memory B cells, CD19^+^/CD3^−^/CD38^++^/IgD^−^ plasma (PC) B cells, CD19^−^/CD3^+^ T cells, and CD19^−^/CD3^−^ non-B/T cells. Symbols indicate median fluorescent intensities (MFI) normalized to negative control experiments (SA-PE only). Median values for 9 independent tonsil specimen are depicted by red bars with numerical values indicated. Statistical significance was determined using a Kruskal–Wallis test with Dunn’s posthoc analysis (*n* = 8). Median values are indicated by red lines, * *p* < 0.05, ** *p* < 0.01, n.s. *p* > 0.05.

Decameric VLRB MM3 allowed for the specific detection of primary human plasma cells [19]. Similar to recombinant decameric VLRB MM3, the inclusion of tetVLRB MM3 coupled to SA-PE in an antibody panel of various directly labeled conventional monoclonal antibodies resulted in strong binding to plasma cells (Figure 4).

**Figure 5:**
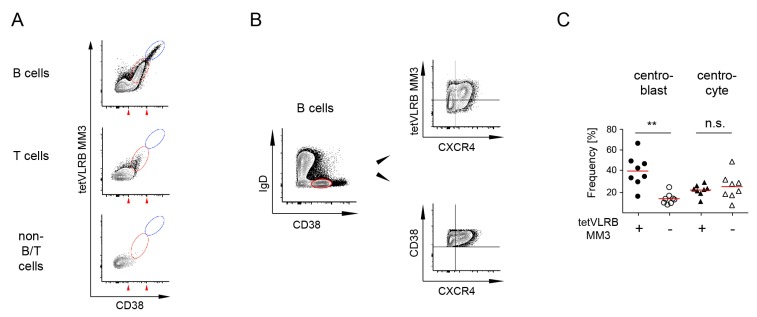
Analysis of tetVLRB MM3 tetramer-reactive germinal center cells. (**A**) Cells located within the blue gate were tetVLRB MM3 reactive plasma cells and were found only among B cells (CD19^+^/CD3^−^) and not among T cells (CD19^−^/CD3^+^) and non-B/T cells (CD19^−^/CD3^−^). Cells with reduced tetVLRB MM3 reactivity (red gate) were detected among B cells but were virtually absent among T and non-B/T cells. Red arrowheads below the *x*-axis indicate the range of CD38 expression of germinal center B cells. (**B**) Analysis of germinal center B cells for tetVLRB MM3 tetramer reactivity. Germinal center B cells were gated based on CD38 expression levels (red gate, assessed using anti-human CD38 antibody, clone HIT-2), separated into CXCR4-positive centroblasts and CXCR4-negative centrocytes and analyzed for tetVLRB MM3 tetramer reactivity. Shown is a representative sample of 8 independent tonsil specimens. (**C**) Frequencies of tetVLRB MM3 reactive GC cell populations. Statistical significance was determined using the Friedman test with Dunn’s posthoc analysis (*n* = 8). Median values are indicated by red lines, ** *p* < 0.01, n.s. *p* > 0.05.

Interestingly, median fluorescent intensities of germinal center (GC) cells were slightly but significantly elevated compared to naïve and memory B cells and non-B/T cells. This GC B cell signal could not be detected in our initial characterization of decameric VLRB MM3 [19]. Displaying tetVLRB MM3 vs. anti-CD38 expression profiles gated on CD19^+^ B cells, CD3^+^ T cells, and CD19^−^/CD3^−^/non-B/T cells revealed the complete absence of tetVLRB MM3 reactive cells among non-B/T cells and only infrequent tetVLRB MM3 reactive events among T lineage cells (Figure 5A). However, this gating strategy revealed a subpopulation of tetVLRB MM3 reactive cells with CD38 expression levels (assessed using anti-human CD38 antibody, clone HIT-2) consistent with GC B cells (Figure 5A). Differential recognition of CD38 by tetVLRB MM3 on a subpopulation of cells with virtually unchanged overall CD38 expression levels prompted us to examine potential differential recognition of GC centrocytes (CC) and centroblasts (CB) by tetVLRB MM3. Using CXCR4 as a marker to discriminate CXCR4^+^ CB from CXCR4^-^ CC [28], we determined that the majority of CB cells were tetVLRB MM3 reactive, albeit at low levels, whereas no differential recognition among CC cells could be detected (Figure 5B, top panel). This differential reactivity to CD38 on CXCR4^+^ CB could not be seen in contour blots displaying CD38 vs. CXCR4 (Figure 5B, bottom panel). These experiments indicated that the increased sensitivity of tetVLRB MM3 compared to conventional decameric VLRB MM3 allowed differential binding analyses to cell populations with lower CD38 expression than CD38^++^ PC (Figure 5C).

## 4. Discussion

The non-conventional VLR antibodies of the evolutionarily distant jawless vertebrates provide a source of anticipatory receptors with structural characteristics distinct from those of conventional mammalian immunoglobulins. Monoclonal VLR antibodies with specificities to carbohydrate antigens [18,29,30,31,32] or tyrosine sulfation-dependent antigen recognition [21] emphasize the potential of VLR antibodies as a novel class of reagents in biomedical research applications. Previously, we isolated the decameric VLRB MM3 antibody whose recognition of human CD38 correlated with dimerization and CD38 enzymatic activity [19]. While these antigen recognition characteristics make decameric VLRB MM3 an important research reagent for studies focusing on CD38, for example, its inclusion in multi-color flow cytometry panels, the inability to directly label this reagent (and other VLRB antibodies) has been an impediment to its ready use in flow cytometry-based experimental designs. To address this challenge, we investigated the generation of VLRB tetramers in an experimental approach used successfully for studies on antigen-specific T cell responses.

VLRB antibodies display avidity-based antigen binding, and monomeric VLRB antigen recognition units typically do not bind antigen [19,20]. Coupling of biotinylated, monomeric VLRB MM3 antigen-binding units provided the necessary avidity for CD38 binding. Using a coumermycin-inducible dimerization system, we could demonstrate that induced CD38 dimerization resulted in increased binding of tetVLRB MM3 tetramers and that pre-incubation with non-hydrolyzable NAD analogs blocked CD38 recognition. These experiments showed that the binding characteristics of VLRB MM3 to human CD38, which correlated with CD38 dimerization and enzymatic activity, were maintained for tetVLRB MM3 tetramers. Furthermore, these experiments indicated that binding of tetVLRB MM3 to CD38 was anticipated to interfere with CD38 enzymatic activity, as we demonstrated for decameric VLRB MM3 [19]. Blocking experiments using non-hydrolyzable NAD analogs indicated that the epitope recognized by tetVLRB MM3 was located within the enzymatic pocket of CD38. This suggested an antigen-recognition modus similar to the recognition of hen egg lysozyme (HEL) by VLRB2.D in which the flexible loop protruding from the capping C-terminal LRR is making contact with residues located within the enzymatic pocket of HEL [17]. Structural analysis of VLRB MM3 antigen-binding units in the complex with CD38 would resolve the mechanism by which VLRB MM3 and the CD38 substrate NAD compete for binding to the receptor.

It was unexpected to observe greatly enhanced sensitivity of tetVLRB MM3 tetramers compared to conventional decameric VLRB MM3 both in cell line and primary cell experiments. Since the 6xHis and HA-epitope tags were engineered into the stalk region of the decameric VLRB MM3 molecule, the increased signal intensities-obtained tetVLRB MM3 tetramers compared to non-tetrameric VLRB MM3 reagents could reflect steric hindrance impeding epitope tag recognition by anti-6xHis or anti-HA antibodies used in the detection of conventional decameric VLRB MM3. Plasma cells were the most strongly recognized cells by tetVLRB MM3 tetramers and conventional decameric VLRB MM3. However, the use of tetVLRB MM3 tetramers in an antibody cocktail used for the analysis of tonsillar B cells revealed that GC centroblasts but not centrocytes preferentially reacted with tetVLRB MM3, indicating the increased occurrence of dimeric CD38 in these GC B cells. Studies on affinity maturation and class switch recombination occurring within the germinal center and differences between the centrocyte and centroblast B cell populations delineated the contributions of antigen receptors, costimulatory receptors, chemokine receptors, and regulatory effects on metabolic pathways in B and T cells [33,34,35]. Increased binding of tetVLRB MM3 to germinal center centroblasts indicated differential CD38 involvement on B lineage cells in the germinal center reaction, a facet of affinity maturation and class switch recombination that remains to be explored. Our study established tetVLRB MM3 as a versatile reagent for the study of human CD38 that complements conventional antibodies targeting CD38.
